# Identification of TSPAN4 as Novel Histamine H_4_ Receptor Interactor

**DOI:** 10.3390/biom11081127

**Published:** 2021-07-30

**Authors:** Xiaoyuan Ma, Eléonore W. E. Verweij, Marco Siderius, Rob Leurs, Henry F. Vischer

**Affiliations:** Division of Medicinal Chemistry, Faculty of Science, Amsterdam Institute of Molecular and Life Sciences, Vrije Universiteit Amsterdam, 1081 HZ Amsterdam, The Netherlands; x.ma@vu.nl (X.M.); noortje.verweij@xs4all.nl (E.W.E.V.); m.siderius@vu.nl (M.S.); r.leurs@vu.nl (R.L.)

**Keywords:** histamine, H_4_R, GPCR, GPCR-interacting proteins, membrane yeast two hybrid, TSPAN4, tetraspanin

## Abstract

The histamine H_4_ receptor (H_4_R) is a G protein-coupled receptor that is predominantly expressed on immune cells and considered to be an important drug target for various inflammatory disorders. Like most GPCRs, the H_4_R activates G proteins and recruits β-arrestins upon phosphorylation by GPCR kinases to induce cellular signaling in response to agonist stimulation. However, in the last decade, novel GPCR-interacting proteins have been identified that may regulate GPCR functioning. In this study, a split-ubiquitin membrane yeast two-hybrid assay was used to identify H_4_R interactors in a Jurkat T cell line cDNA library. Forty-three novel H_4_R interactors were identified, of which 17 have also been previously observed in MYTH screens to interact with other GPCR subtypes. The interaction of H_4_R with the tetraspanin TSPAN4 was confirmed in transfected cells using bioluminescence resonance energy transfer, bimolecular fluorescence complementation, and co-immunoprecipitation. Histamine stimulation reduced the interaction between H_4_R and TSPAN4, but TSPAN4 did not affect H_4_R-mediated G protein signaling. Nonetheless, the identification of novel GPCR interactors by MYTH is a starting point to further investigate the regulation of GPCR signaling.

## 1. Introduction

Histamine is a key mediator of allergic inflammation and is released from mast cells and basophils upon allergen binding. The histamine H_4_ receptor (H_4_R) is predominantly expressed on immune cells and mediates histamine-induced chemotaxis and production of inflammatory cytokines [1,2]. Importantly, H_4_R-deficient mice revealed a role of this receptor in pruritus, dermatitis, asthma, and arthritis disease models [2]. Consequently, the H_4_R has been recognized as potential anti-inflammatory drug target, and selective antagonists are currently in clinical trials to counteract atopic dermatitis, psoriasis, allergic rhinitis, bronchial allergen challenge, asthma, and rheumatoid arthritis [2].

The H_4_R is a G protein-coupled receptor (GPCR) that activates heterotrimeric Gα_i_ protein-mediated intracellular signal transduction resulting in decreased cAMP production [3,4,5,6,7], increased Ca^2+^ mobilization [8,9,10], activation of extracellular-signal regulated kinase (ERK)1/2 [11,12,13,14,15], as well as cytoskeletal changes [8]. Similar to most other GPCRs, the H_4_R can interact with G protein receptor kinases (GRKs) and β-arrestins that are involved in receptor internalization and consequently the regulation of agonist-induced G protein signaling [14,15].

GPCRs have been found to also interact with other membrane and/or intracellular proteins in addition to heterotrimeric G proteins, GRKs, and β-arrestins. These so-called GPCR interacting proteins (GIPs) can mediate and/or modulate GPCR signaling and/or trafficking [16,17,18], and have been identified by various biochemical (e.g., pull-downs from cell lysates using Glutathione S-transferase (GST) or His6-tagged GPCR C-tails [19,20], or HDL-reconstituted GPCR [21]), cell biological (e.g., ascorbate peroxidase (APEX)-catalyzed proximity labeling in living cells followed by affinity chromatography, enzyme digestion, and mass spectrometric analysis [22,23,24]), and genetic approaches (e.g., membrane yeast two-hybrid (MYTH) [25,26,27,28,29,30,31]).

**Figure 1:**
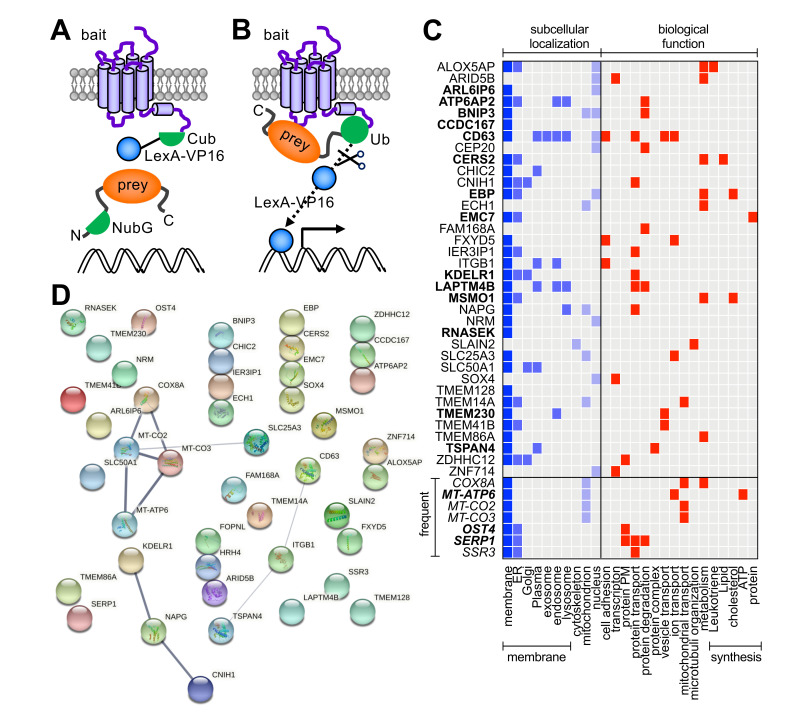
Schematic overview of the split-ubiquitin membrane-based yeast two-hybrid (MYTH) assay. (**A**) Diploid yeast co-expressing the membrane targeted H_4_R-Cub-LexA-VP16 bait and cytosolic (depicted) or membrane-associated N-terminally tagged NubG prey proteins was generated by mating of haploid yeasts expressing the individual bait and prey constructs. (**B**) Interaction of the H_4_R-Cub-LexA-VP16 ‘bait’ with NubG-protein ‘prey’ results in functional reconstitution of a pseudo-ubiquitin, which is subsequently recognized by cytosolic ubiquitin-specific proteases leading to the cleavage of the LexA-VP16 transcriptional activator and the expression of (*HIS3*, *ADE2*, *LacZ*) reporter genes. (**C**) Subcellular localization and biological function summary of the 43 hits were retrieved from the Uniprot database (https://www.uniprot.org; accessed on 24 June 2021). Gene names are indicated and full description of proteins with Uniprot codes are presented in Supplementary Table S1. Hits in bold have been found to interact with other GPCRs in MYTH screens (see Supplementary Table S1), whereas frequent MYTH screen hits are indicated in italics. ER = endoplasmic reticulum; PM = post-translational modification (**D**) STRING analysis (v.11.0; https://string-db.org; accessed on 25 June 2021) of hits from the MYTH screen of H_4_R (bait) on an unstimulated Jurkat T cell DUALmembrane cDNA library. Known interactions between proteins in the STRING database are indicated by connecting lines. The thickness of the line represents the degree of confidence prediction of the interaction.

In this study, the MYTH approach was used to identify GIPs of the human H_4_R from an unstimulated Jurkat T cell cDNA library [32]. In short, the H_4_R was fused at the N-terminus to the yeast STE2 leader sequence to target the fusion protein to the membrane of yeast, whereas the C-terminal half of ubiquitin (Cub) and the LexA-VP16 transcriptional activator were fused in frame to the intracellular C-terminal tail of H_4_R. The prey proteins encoded by the naïve Jurkat T cell cDNA library were N-terminally tagged with the N-terminal half of ubiquitin that contains an isoleucine 13 to glycine mutation (NubG) to prevent spontaneous ubiquitin reconstitution (Dualsystems Biotech, Switserland) (Figure 1A). Interaction of unliganded H_4_R-Cub-LexA-VP16 (bait) with NubG-tagged prey proteins in yeast allows reconstitution of a pseudoubiquitin moiety, which is subsequently recognized by endogenous ubiquitin-specific proteases resulting in the cleavage of the LexA-VP16 transcriptional regulator. The released transcriptional regulator translocates into the nucleus and activates transcription of LexA-promoter fused reporter genes for selection (*LacZ*, *HIS3*, *ADE2*) (Figure 1B).

This MYTH approach identified 43 novel GIPs for the H_4_R. The interaction of one potential GIP (i.e., human tetraspanin 4; TSPAN4) was further validated in transfected HEK293T cells using bioluminescence resonance energy transfer (BRET), bimolecular fluorescence complementation (BiFC), and co-immunoprecipitation. In addition, the consequence of this H_4_R-TSPAN4 interaction was evaluated in ligand binding and H_4_R-mediated G protein activation assays.

## 2. Materials and Methods

### 2.1. Materials and Reagents

Fetal bovine serum was obtained from Bodinco (Alkmaar, The Netherlands), and penicillin/streptomycin was purchased from GE Healthcare (Uppsala, Sweden). Dulbecco’s Modified Eagle’s Medium (DMEM, #41966-029), Dulbecco’s phosphate-buffered saline (DPBS, #D8662), Hanks’ Balanced Salt Solution (HBSS, #14025-050), trypsin-EDTA, Pierce^TM^ bicinchoninic acid (BCA) protein assay kit, GeneJET gel extraction kit, and GenJET plasmid Miniprep kit were purchased from Thermo Fisher Scientific (Waltham, MA, USA). [^3^H]-histamine (specific activity 20.0 Ci/mmol), Microscint-O scintillation liquid, GF/C filter plates were purchased from PerkinElmer (Groningen, The Netherlands). NanoGlo^®^ was bought from Promega (Madison, WI, USA). 4′,6-diamidino-2-phenylindole (DAPI), Whatman^®^ Westran^®^ PVDF membranes, cOmplete™, EDTA-free Protease Inhibitor Cocktail and histamine·2HCl were bought from Sigma-Aldrich (St. Louis, MO, USA). Linear polyethylenimine (PEI, 25-kDa) was obtained from Polysciences (Warrington, FL, USA). Monoclonal Anti-HA (rat) antibody (#11867423001) was obtained from Roche (Roche Diagnostics; Mannheim, Germany). Goat polyclonal antibody to Venus (#orb334993) was bought from Biorbyt Ltd. (Cambridge, UK). Goat anti-Rat IgG (H+L) Secondary Antibody and Rabbit anti-Goat IgG (H+L) Secondary Antibody were from Bio-Rad Laboratories (Hercules, CA, USA). All other reagents were of analytical grade and obtained from conventional commercial sources.

### 2.2. MYTH Constructs and Screen

The MYTH bait vector pBT3-STE-H_4_R was generated by PCR. A *SfiI* fragment containing the full open reading frame of H_4_R was subcloned in frame downstream of the STE2 leader sequence and upstream of the sequence encoding for Cub (i.e., amino acids 34–76 of ubiquitin) a GG-containing linker and the LexA-VP16 transcriptional regulator in the pBT3-STE bait vector that contains a *LEU2* marker (Dualsystems, Switzerland). The DUALmembrane prey library from Dualsystems (catalog # P02205) consists of cDNA sequences from unstimulated Jurkat T cell containing ~9 × 10^6^ independent clones, with an average size of 1.5 kb, that were N-terminally fused to NubG in the *TRP1* marker-containing pDSL-Nx prey vector (Dualsystems, Switzerland). Hits from the H_4_R MYTH screen were analyzed using Uniprot database (https://www.uniprot.org; accessed 24 June 2021) [33] and the protein–protein network tool STRING (v11.0; https://string-db.org; accessed on 25 June 2021) [34].

### 2.3. MYTH Strains and Growth Conditions

Yeast cells were routinely grown at 30 °C in synthetic yeast nitrogen base (YNB) medium (0.67% yeast nitrogen base, 2% glucose) supplemented with the required amino acids in liquid cultures or on 2% agar plates. Haploid *Saccharomyces cerevisiae* strains (Dualsystems Biotech, Switserland) THY.AP4 (genotype: Mata, *ura3*, *leu2*, *LexA::LacZ::trp1*, *LexA::HIS3*, *LexA::ADE2*) and THY.AP5 (genotype: Matα, *URA3*, *leu2*, *trp1*, *his3 loxP::ade2*) were transformed with ‘bait’ pBT3-STE2-H_4_R-*LEU2* and pDSL-Nx-Prey-*TRP1* vectors, respectively, using the freeze–thaw transformation method [35]. In addition, THY.AP4 and THY.AP5 were transformed with plasmids pMETYC-*LEU2* (i.e., empty Cub) and pNubWT-*TRP1* (i.e., wild type Nub) or pXN21-*TRP1* (i.e., empty Nub) for MYTH control experiments. Next, yeast mating was performed by mixing THY.AP4 and THY.AP5 haploids on YDP plates (1% Yeast extract, 2% bacto-peptone, 2% glucose, 2% agar) and incubation ON at 30 °C. Diploids were selected on YNB medium lacking leucine and tryptophane to verify presence of both ‘bait’ and ‘prey’ vectors. Interaction between the bait H_4_R-Cub-LexA-VP16 and N-terminally NubG-tagged prey proteins was measured as growth in liquid cultures starting at OD600 = 0.1. (Infinite 200PRO plate reader, Tecan, Germany) and on agar plates lacking leucine, tryptophane (selection diploids) and histidine (*LexA::HIS3* expression induced by LexA-VP16).

### 2.4. Cell Culture and Transfection

HEK293T cells (ATCC; Manassas, VA, USA) were cultured in DMEM (Dulbecco’s modified Eagle’s medium) supplemented with 10% FBS and 1% penicillin/streptomycin (50 μg/mL) at 37 °C, 5% CO_2_. Cells (2 × 10^6^) were seeded in a 10 cm dish and transiently transfected the next day with indicated amounts of DNA plasmids using 20 μg 25 kDa linear polyethylenimine (PEI), as previously described. Empty pcDEF3 plasmid was used to keep total DNA amounts at 5 μg for each transfection. For saturation BRET experiments by gene-dosing, 2 × 10^4^ cells/well were seeded in 0.1% poly-L-lysine-coated white bottom 96-well plates and transiently transfected the next day using 25 kDa linear PEI, as previously described [36].

### 2.5. Mammalian Expression Constructs

The human H_4_R-Nluc fusion (H_4_R; NM_021624.3) construct was generated by subcloning H_4_R-Rluc8 into H_1_R-Nluc/pcDEF3 using flanking *Kpn*I and *Spe*I restriction sites, as previously described [14,37]. Human TSPAN4 (TSPAN4; NM_003271.4) was genetically fused to mVenus at the intracellular N- or C-terminus by substituting start or stop codon, respectively, with *Spe*I-*Not*I restriction sites (coding for TSAAA linker) using PCR as previously described [14]. The H_4_R fusion to the N-terminal split-fragment of mVenus (V_1_: amino acids 1-155) in pcDEF3 was previously reported [38], whereas the C-terminal split-fragment of mVenus (V_2_: amino acids 156-240) was genetically fused to the N-terminus of TSPAN4 via the aforementioned TSAAA-linker sequence using PCR and subsequent subcloned into pcDNA3.1 or pcDEF3 using the introduced restriction enzymes. HA-H_4_R in pcDEF3 was previously described [39]. Gα_i2_ protein biosensor plasmid was kindly provided by Dr. Schihada (Karolinska Institutet, Department of Physiology and Pharmacology, Stockholm, Sweden) [40]. All constructs were verified by DNA sequencing.

### 2.6. Bioluminescence Resonance Energy Transfer (BRET)-Based Close Proximity Detection

HEK293T cells were transiently transfected with 12.5 ng H_4_R-Nluc/pcDEF3 in combination with 0 to 500 ng mVenus-TSPAN4/pcDNA3.1 or TSPAN4-mVenus/pcDEF3 plasmids per 10^6^ cells in white-bottom 96-well plates. Forty-eight hours after transfection, medium was aspirated from the cells and replaced by assay buffer Hank’s Balanced Salt Solution (HBSS). Luminescence (lum) was measured in time upon stimulation with vehicle or 10 µM histamine in the presence of Nanoglo (3.2 µL/mL) at 37 °C using the Mithras LB940 multimode microplate reader (Berthold, Germany) at 540–40 nm and 480–20 nm. The expression of mVenus-TSPAN4 or TSPAN4-mVenus was measured as fluorescence (fluo) at 540 nm emission upon excitation at 485 nm in the Mithras LB940 plate reader. The BRET ratio signal was calculated as the 540 lum/480 lum emission ratio and presented as function of the mVenus/Nluc expression levels as calculated by 540 fluo/480 lum ratio [41].

Saturation BRET curves were fitted using the nonlinear One site-specific binding model in GraphPad Prism 8.0. For histamine concentration-response curves, cells were co-transfected in 10 cm dishes with 100 ng H_4_R-Nluc/pcDEF3 and 2 µg mVenus-TSPAN4/pcDNA3.1 or TSPAN4-mVenus/pcDEF3 plasmids and transferred the next day into white-bottom 96-well plates (5 × 10^4^ cells/well). Two days after transfection, BRET signal was measured in the presence of increasing concentration histamine at 37 °C.

### 2.7. Biomolecular Fluorescence Complementation (BiFC)-Based Close Proximity Detection

HEK293T cells were co-transfected with 0.5 μg H_4_R-V1/pcDEF3 and 0.5 μg V2-TSPAN4/pcDNA3.1 per dish. The next day, 8 × 10^5^ cells/well were transferred on 0.1% poly-L-lysine-coated cover slides in 6-well plates. Forty eight hours post-transfection, the cells were stimulated with vehicle or 10 µM histamine for 30 min at room temperature. After fixation by 4% paraformaldehyde (PFA), the cells were stained with DAPI for nuclear staining and the reconstituted green fluorescence were visualized with an Olympus FSX-100 microscope at 475/30 nm excitation and 535/30 nm emission.

### 2.8. Co-Immunoprecipitation

HEK293T cells were transfected with 100 ng HA-H_4_R/pcDEF3 and 500 ng mVenus-TSPAN4/pDNA3.1 plasmids per dish. Forty-eight hours after transfection, the cells were solubilized in RIPA buffer (150 mM NaCl, 1% NP-40, 1 mM EDTA, 1 mM CaCl_2_, 10% glycerol) for 2 h at 4 °C in the presence of protease inhibitor (Roche, fresh on day of use) The solubilized cells were centrifuged at 15,000× *g* for 10 min at 4 °C, then 30 µL of supernatant was stored for lysis detection. The remaining supernatant was then incubated overnight together with 50 µL suspension of agarose-conjugated monoclonal Anti-HA antibody (#A2095, Sigma-Aldrich) at 4 °C with constant agitation. The next day, the agarose beads were washed three times with wash buffer (0.1% TritonX100, 50 mM Tris pH 7.4, 300 mM NaCl, 5 mM EDTA), and the co-immunoprecipitated samples were eluted using 6X SDS Sample Buffer (0.375 M Tris pH 6.8, 12% SDS, 60% glycerol, 0.6 M DTT, 0.06% bromophenol blue). Next, eluent and lysis samples were loaded on a 10% SDS-PAGE gel for electrophoresis, followed by transfer to 0.45 µM PVDF membranes and blocking by 5% slim milk for 2 h incubation at room temperature. Next, the membranes were incubated with primary antibodies anti-HA (rat) (1:1000 in 5% BSA/TBST) or anti-Venus (goat) (1:2000 in 5% BSA/TBST) overnight incubation at 4 °C, followed by secondary antibodies Goat anti-Rat IgG-HRP conjugate (1:5000 in 5% slim milk) or Rabbit anti-Goat IgG-HRP conjugate (1:5000 in 5% slim milk), respectively, for 2 hrs incubation at room temperature. The immunoreactive bands were detected using Pierce ECL Western Blotting Substrate (Thermo Fisher Scientific, Waltham, MA, USA) and visualized with Chemidoc^TM^ (Bio-Rad).

### 2.9. [^3^H]Histamine Binding Assay

HEK293T cells were transiently transfected with 100 ng H_4_R-Nluc and/or 2 µg mVenus-TSPAN4 or TSPAN4-mVenus plasmids and collected after two days, as previously described [7]. The cells were homogenized in binding buffer (50 mM Tris-HCl, pH 7.4) and incubated with increasing concentrations of [^3^H]histamine (0–40 nM) in duplicate for 2 h at 25 °C in the presence and absence of 50 μM JNJ7777120 to detect total and nonspecific binding, respectively. Incubation was stopped by rapid filtration through 96-well GF/C plates that were pre-soaked with 0.5% (*v*/*v*) PEI, using a 96-well Filtermate harvester (PerkinElmer, Groningen, The Netherlands). Next, the GF/C filter plates were rapidly washed four times with ice-cold wash buffer (50 mM Tris-HCl, pH 7.4, 4 °C) and dried at 52 °C for at least 30 min before addition of 25 μL/well Microscint-O to quantify radioactivity using the Microbeta Wallac Trilux scintillation counter (Perkin-Elmer). Protein concentration of the cell homogenates was measured by a BCA protein assay, according to the manufacturer’s recommendation. Binding affinity and B_max_ values were determined using the One site-total and nonspecific binding model in GraphPad Prism 8.0.

### 2.10. BRET-Based G Protein Activation Biosensor

HEK293T cells were co-transfected with 100 ng HA-H_4_R/pcDEF3 and 1.5 µg tricistronic Gα_i2_ biosensor (G_β1_-T2A-cpVenus-G_γ2_-IRES-Gα_i2_-Nluc) plasmids per dish with or without 500 ng V2-TSPAN4/pcDNA3.1. The next day, 5 × 10^4^ cells/well were transferred on 0.1% poly-L-lysine-coated white-bottom 96-well plates. Two days after transfection, BRET signal was measured at 37 °C in response to histamine stimulation for 20 min, as described above.

### 2.11. Data Analysis

Regression and statistical analyses of experimental data was performed by GraphPad Prism 8.0 (GraphPad Software, San Diego, CA, USA). ImageJ software (National Institutes of Health, Bethesda, MD, USA) was used for Western blot quantification.

## 3. Results

### 3.1. Identification of H_4_R Interactors by MYTH Screen of Jurkat T Cell cDNA Library

To identify H_4_R interactors, a MYTH screen was performed by Dualsystems Biotech AG (Schlieren, Switzerland) using their unstimulated Jurkat T cell DUALmembrane cDNA library. Diploid yeast clones that grew under selection were picked from primary screening plates and transferred to liquid screening medium. Clones were passaged for five rounds to eliminate non-specific interactors and subsequently assayed for the *lacZ* reporter gene using a β-galactosidase assay. Screening of 2.5 × 10^6^ transformants yielded 43 potential interactors of H_4_R (Figure 1C; Supplementary Table S1). Seven (16%) of these interactors (i.e., MT-ATP6, MT-CO2, MT-CO3, COX8A, SSR3, OST4, and SERP1) were recognized as highly connected proteins that are hits in the majority of MYTH screens by Dualsystems. The 43 hits were analyzed using the STRING protein-protein association network analysis tool but did not reveal known interconnections between bait (i.e., HRH4) and identified prey proteins (Figure 1D). The relative abundance of membrane (-associated) proteins (84%) amongst the prey proteins seems evident and the presence of two members of the membrane-associated tetraspanin family (CD63, TSPAN4) was noted (Figure 1C; Supplementary Table S1).

### 3.2. MYTH between H_4_R and Tetraspanins TSPAN4 and CD63 Is Decreased by Histamine Stimulation

We have selected CD63 and TSPAN4 for in-house MYTH validation as tetraspanins have been reported in immune cells to act as scaffolds for signaling cascade components, mediate cell–cell communication, and are involved in cellular migration [42,43].

To this end, we used the STE2-H_4_R-Cub-LexA-VP16 construct as bait and validated its expression and potential to interact with Nub-tagged baits. The bait construct was validated, generating diploid strains carrying either wild type Nub expressing vector (pNub wt) or empty vector (pXN21) as prey plasmids. Besides growing on the -leu-trp medium, selecting for diploids, the positive control displayed growth on media without histidine, indicative of interaction between bait and prey (Supplementary Figure S1).

Validation of the prey plasmids was made by generating diploids either with an empty bait plasmid (pMETYc) or the H_4_R-Cub-LexA-VP16 bait plasmid. The diploids with the pMETYc negative control as bait only grew on media with histidine, whereas the H_4_R-TSPAN/CD63 combinations displayed growth on selective media without histidine (Supplementary Figure S1).

**Figure 2:**
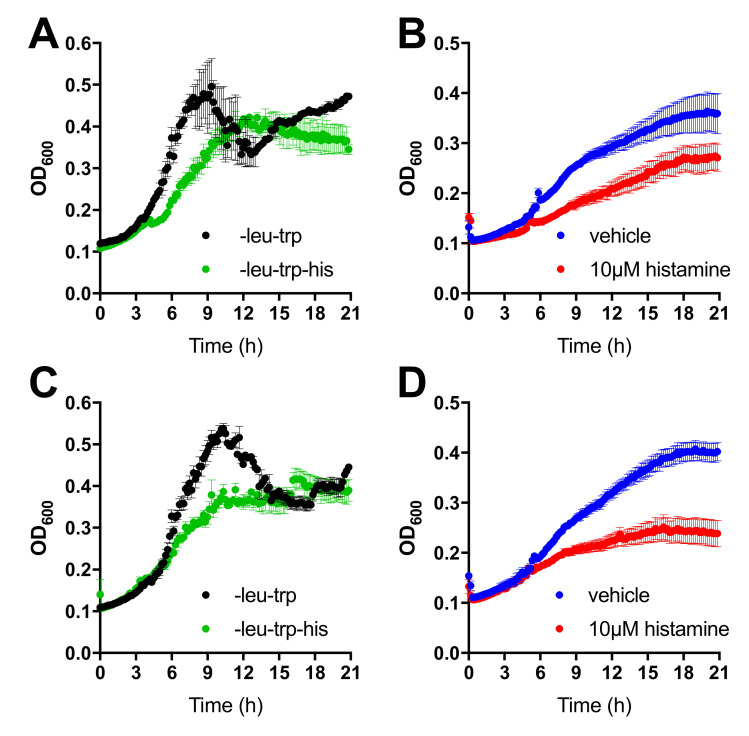
Interaction between H_4_R with TSPAN4 and CD63 as measured by MYTH liquid growth assay. (**A**) Co-expression of the H_4_R-Cub-LexA-VP16 and NubG-TSPAN4 constructs is evidenced by growth in media without leucine and tryptophane (black trace, selecting for diploids). Two-hybrid interaction and subsequent expression of the *HIS3* reporter gene results in growth on media additionally depleted for histidine (green trace, selecting for a two-hybrid interaction). (**B**) Modulation histidine-independent growth of the H_4_R-Cub-LexA-VP16 and NubG-TSPAN4 expression diploid by vehicle (blue trace) or 10 µM histamine (red trace). (**C**) Interaction between H_4_R-Cub-LexA-VP16 and NubG-CD63 as describe in (**A**). (**D**) Modulation of the interaction between H_4_R (bait) and CD63 (prey) as described in (**B**). Data are displayed as mean ± SEM from 3 independent growth traces.

Two-hybrid interactions between H_4_R-Cub-LexA-VP16 and NubG-TSPAN4 (Figure 2A) and NubG-CD63 (Figure 2C) were confirmed as growth in liquid media. Again, the diploid strains expressing bait and prey are able to grow in media without histidine, indicative of the interaction between H_4_R and TSPAN4 and CD63, respectively. Next, we examined the possibility to modulate these interactions by addition of 10 µM histamine. Growth of the diploids expressing the H_4_R-Cub-LexA-VP16 as bait in combination with NubG-TSPAN4 (Figure 2B) or NubG-CD63 (Figure 2D) was diminished by histamine, suggesting reduced interactions.

### 3.3. Saturation BRET Confirms Interaction between H_4_R and TSPAN4 in HEK293T Cells and Can Be Reduced by Histamine Stimulation

**Figure 3:**
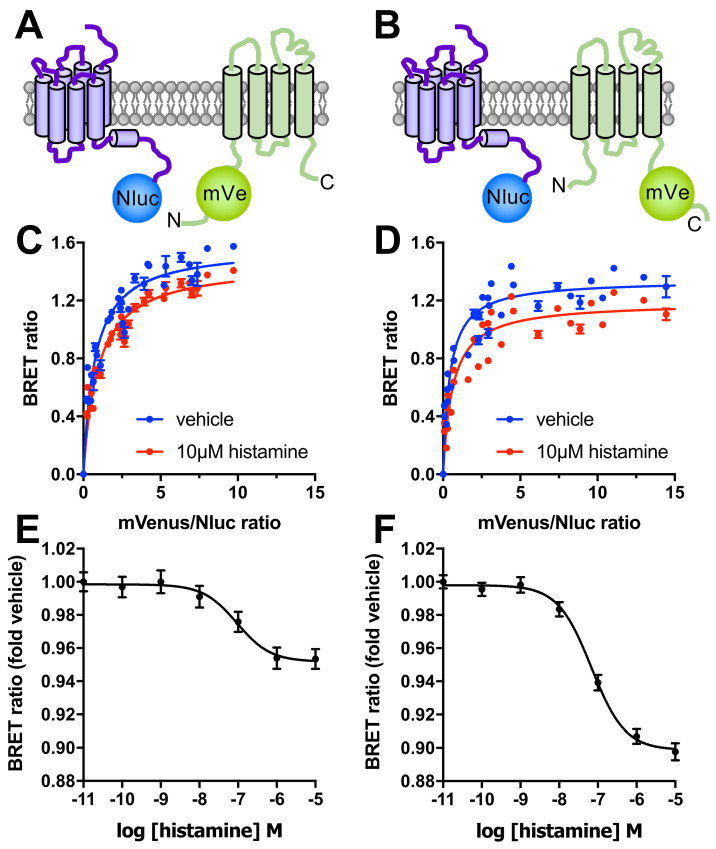
Detection of close proximity between H_4_R and TSPAN4 in HEK293T cells by BRET. (**A**,**B**) In frame fusion of the bioluminescent enzyme to the C-terminus of H_4_R and mVenus to either the intracellular N- or C-terminal tail (panels **A**,**B**, respectively) of TSPAN4 allows BRET detection of their proximity in cells co-expressing these constructs. (**C**,**D**) Saturation BRET curves measure the BRET ratio between H_4_R-Nluc and either (**C**) mVenus-TSPAN4 or (**D**) TSPAN4-mVenus as function of increasing mVenus/Nluc ratios under basal conditions (vehicle in blue) and upon stimulation with 10 µM histamine (red). Saturation BRET data are displayed as mean ± SD from 3 independent experiments performed in triplicate (**E**,**F**) Effect of increasing concentrations histamine on the basal interaction between H_4_R-Nluc and either (**E**) mVenus-TSPAN4 or (**F**) TSPAN4-mVenus. Data are displayed as mean ± SEM from 5 independent experiments performed in triplicate.

To monitor the interaction between H_4_R and TSPAN4 in mammalian cells by bioluminescence resonance energy transfer (BRET), the bioluminescent enzyme NanoLuc (Nluc) was fused in-frame to the intracellular C-terminal tail of H_4_R, whereas the fluorescent protein mVenus was fused to either the intracellular N- or C-terminus of TSPAN4 (Figure 3A,B, respectively).

Co-transfection of HEK293T cells with a constant amount of H_4_R-Nluc (i.e., BRET-donor) in combination with increasing amounts of either mVenus-TSPAN4 or TSPAN4-mVenus (i.e., BRET-acceptors) plasmids resulted in hyperbolic BRET signals (Figure 3C,D, respectively). These saturable BRET signals suggest that Nluc and mVenus are brought in close proximity (<10 nm) as a consequence of specific interactions between H_4_R and TSPAN4 in transfected HEK293T cells rather than random collisions [41].

The BRET_max_ values (1.56 ± 0.18 and 1.36 ± 0.06) were comparable (Student’s *t*-test; *p* > 0.05) between mVenus-TSPAN4 and TSPAN4-mVenus, respectively, when co-expressed with H_4_R-Nluc (Figure 3C,D, respectively). Moreover, BRET_50_ values (0.79 ± 0.46 and 0.45 ± 0.18) representing the mVenus/Nluc ratios that produce 50% BRET_max_ signals were also similar (Student’s *t*-test; *p* > 0.05) between both N- and C-terminal fusions of TSPAN4 to mVenus, respectively, indicating a similar propensity of both TSPAN4 fusion constructs to interact with H_4_R-Nluc. These BRET data confirm the basal interaction between H_4_R-Cub-LexA-VP16 and NubG-TSPAN4 that was observed in the MYTH assay.

Stimulation of the transfected HEK293T cells with 10 μM histamine for 30 min resulted in a small but consistent reduction (<10%) in BRET_max_ between H_4_R-Nluc and mVenus-TSPAN4 or TSPAN4-mVenus (BRET_max_ = 1.41 ± 0.22 and 1.20 ± 0.06, respectively), without affecting the BRET_50_ values as compared to vehicle-stimulated cells (BRET_50_ = 0.92 ± 0.56 and 0.68 ± 0.37, respectively; Student’s *t*-test; *p* > 0.05). This confirms the observed reduction in yeast growth assays upon histamine stimulation, suggesting that the decrease in BRET is the consequence of reduced H_4_R-TSPAN4 interaction rather than a change in complex conformation resulting in a changed orientation of the BRET donor and acceptor. Histamine reduced the BRET between H_4_R-Nluc and mVenus-TSPAN4 (expression ratio mVenus/Nluc ratio ~6.3, see Figure 3C) or TSPAN4-mVenus (expression ratio mVenus/Nluc ratio ~ 13, see Figure 3D) with similar pEC_50_ values (7.1 ± 0.35 and 7.2 ± 0.15, respectively) (Figure 3E,F).

### 3.4. BiFC Microscopy Reveals H_4_R-TSPAN4 Complexes in HEK293T Cells

**Figure 4:**
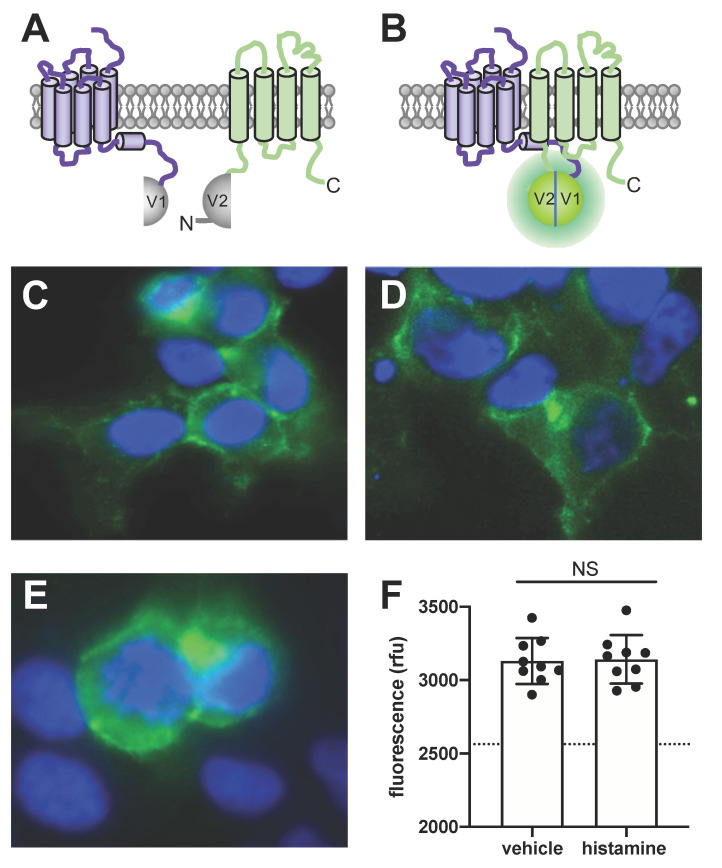
Detection of close proximity between H_4_R and TSPAN4 at the surface of HEK293T cells using bimolecular fluorescence complementation. (**A**,**B**) In-frame fusion of the nonfluorescent complementary mVenus fragments V1 and V2 to the intracellular C-terminal tail of H_4_R and N-terminal tail of TSPAN4 allows functional reconstitution of mVenus that is driven by the close proximity between H_4_R and TSPAN4. (**C**,**E**) HEK293T cells transiently co-transfected with H_4_R-V1 and V2-TSPAN4. Two days post-transfection, the cells were observed under microscope upon stimulation with vehicle (**C**) or 10 µM histamine (**E**). Interaction between H_4_R-V1 and V2-TSPAN4 reconstitutes mVenus (green), nuclei are stained with 4’6-diamidino-2-phenylindole (blue). (**D**) Image of cells two days after transient transfection with mVenus-TSPAN4. Representative images (**C**–**E**) are shown from three independent experiment performed in triplicate. (**F**) Fluorescence intensity of the reconstituted mVenus upon stimulation with vehicle or 10 µM histamine was measured by plate reader at 497-15 excitation and 540-20 emission. Dotted line represents the background fluorescence in mock transfected cells. Data are displayed as mean ± SD from 3 independent experiments performed in triplicate. Statistical difference was analyzed using Student’s *t*-test. NS = no significant difference.

To confirm the close proximity between H_4_R and TSPAN4 in HEK293T cells, we fused the N-terminal fragment of mVenus in frame to the intracellular C-terminal tail of H_4_R (i.e., H_4_R-V1) and the C-terminal fragment of mVenus to the intracellular N-terminal tail of TSPAN4 (i.e., V2-TSPAN4) to monitor bimolecular fluorescence complementation (BiFC) of these mVenus fragments that is driven by the interaction by the proteins to which they are fused (Figure 4A,B), as previously described for complexes between GPCRs [38,44]. Indeed, co-expression of H_4_R-V1 and V2-TSPAN4 in HEK293T resulted in BiFC (Figure 4C), which matches the localization of mVenus-TSPAN4 (Figure 4D). BiFC detection of protein–protein interactions by mVenus reconstitution is known to be virtually irreversible and as anticipated no decrease in fluorescence was observed upon histamine stimulation (Figure 4E,F) [45,46]. In addition, histamine seemed not to affect the localization of the H_4_R-TSPAN4 complexes as compared to vehicle-stimulated cells.

### 3.5. TSPAN4 Co-Immunoprecipitates with H_4_R from Co-Expressing HEK293T Cells

**Figure 5:**
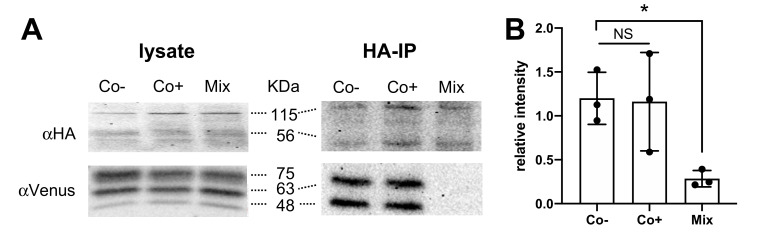
Detection of physical interaction between H_4_R and TSPAN4 in HEK293T cells using co-immunoprecipitation. (**A**) HEK293T cells transiently co-expressing HA-H_4_R and mVenus-TSPAN4 were collected and solubilized upon stimulation with vehicle (Co-) or 10 μM histamine (Co+), whereas cells expressing either HA-H_4_R or mVenus-TSPAN4 were collected and mixed (Mix; 1:1) before solubilization. Lysates were immunoprecipitated (IP) using anti-HA agarose beads and both lysates and IP samples were resolved by SDS-PAGE and immunoblotted using anti-HA (top panel) or anti-Venus (bottom panel) antibodies. Immunoblots are representative of three independent experiments. (**B**) Densitometric analysis (mean ± SEM) of mVenus-TSPAN4 immunoblots by dividing the intensity of co-immunoprecipitated (IP) mVenus (indicated as 48 KDa and 63 KDa) by lysis mVenus (indicated 48 KDa, 63 KDa, and 75 KDa) intensity from three independent experiments using ImageJ software (National Institutes of Health, Bethesda, MD, USA). Statistical difference versus Co- was analyzed using one-way ANOVA followed by Dunnett’s multiple comparison test and indicated by an asterisk *p* < 0.05. NS = no significant difference.

To confirm the physical interaction between H_4_R and TSPAN4, HEK293T cells were transfected with HA-H_4_R and mVenus-TSPAN4, and cell lysates were subjected to immunoprecipitation using an anti-HA agarose beads. Both lysates and immunoprecipitates were resolved by SDS-PAGE and subsequently immunoblotted using rat anti-HA and goat anti-mVenus antibodies. To verify that co-immunoprecipitated H_4_R-TSPAN4 complexes are not the consequence of non-specific aggregation during the cell solubilization procedure, cells transfected with either HA-H_4_R or mVenus-TSPAN4 were mixed prior to the solubilization step. HA-H_4_R and mVenus-TSPAN4 were detected in immunoblots of both co-expressing (Co-) and mixed (Mix) cells (Figure 5A left panel), whereas mVenus-TSPAN4 was only detected in co-expressing cells (Co-) upon immunoprecipitation of HA-H_4_R confirming their physical interaction (Figure 5A right panel). Stimulation of these co-expressing cells (Co+) with 10 µM histamine did not seem to affect the co-immunoprecipitation of TSPAN4 with H_4_R (Figure 5A,B), which is in line with the only small decrease of basal BRET and MYTH.

### 3.6. Overexpression of TSPAN4 Does Not Affect Histamine Binding to H_4_R or Signaling

To evaluate whether the basal interaction of the TSPAN4 affect H_4_R functioning, histamine binding and signaling was evaluated. Co-expression of mVenus-TSPAN4 or TSPAN4-mVenus did not significantly affect the binding affinity of radiolabeled [^3^H]histamine for H_4_R as compared to cell homogenates expressing only H_4_R (Table 1; Supplementary Figure S2A–C). The 5.5-fold reduced total number of H_4_R (B_max_) in cells co-transfected with TSPAN4 as compared to cells expressing only H_4_R is most likely the consequence of overexpression of TSPAN4 resulting in an inefficient transcription and translation of H_4_R (Table 1).

**Figure 6:**
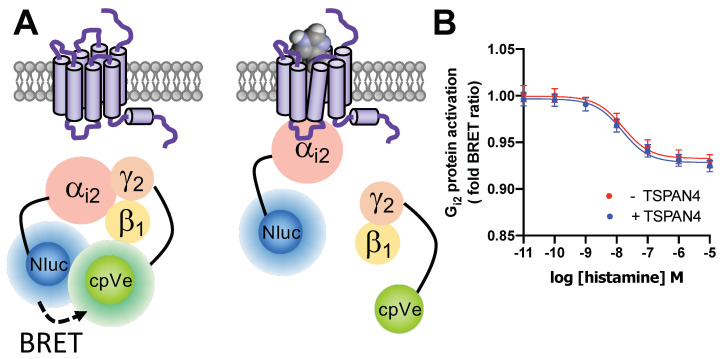
Detection of Gα_i_ protein activity in HEK293T cells by BRET-based G protein biosensor. (**A**) Fusion of Nluc to Gα_i2_ subunit and cpVenus to Gγ subunit allows BRET detection of the heterotrimeric G protein complex. Agonist binding to the H_4_R leads to activation and dissociation of heterotrimeric G proteins, resulting in reduced BRET signal. (**B**) BRET measurements in HEK293T cells transiently co-expressing HA-H_4_R and G protein biosensor (red trace) or HA-H_4_R, V2-TSPAN4 and G protein biosensor (blue trace), upon stimulation with histamine for 20 min. Data are displayed as mean ± SEM from 3 independent experiments performed in triplicate.

Co-expression of TSPAN4 did also not affect H_4_R-mediated heterotrimeric Gα_i2_ protein activation in response to histamine (pEC_50_ = 7.8 ± 0.13) as compared to cells expressing only H_4_R (pEC_50_ = 7.8 ± 0.03) as detected by BRET-based G protein activation sensor (Figure 6A,B).

## 4. Discussion

In the last decade, studies have shown that GPCRs can interact with numerous membrane-associated and cytosolic protein in addition to their well-known coupling to heterotrimeric G proteins, GRKs, and β-arrestins [21,25,26,27,28,29,30,31]. Indeed, GPCR signaling is primarily mediated via the heterotrimeric G proteins, whereas GRKs and β-arrestins play key functions in regulating the duration of G protein signaling. These so-called GPCR-interacting proteins (GIPs) have been found to modulate GPCR activity by for example anchoring GPCRs with their signaling partners in subcellular membrane compartments (e.g., lipid rafts) for directional signaling (e.g., cell migration), or forming signalosome complexes to signal in G protein-independent manner [16,17,18]. In contrast to most G proteins, GRK, and β-arrestin, GIPs are often less widely expressed allowing cell-type specific fine-tuning of GPCR activity [16].

The MYTH approach has been successfully used to screen a human fetal brain DUALmembrane NubG-X cDNA library using ~50 other GPCRs as bait and yielding ~700 potential GIPs [26,27,29], whereas validated GIPs have also been identified in MYTH screen using human liver and pancreatic islets cDNA libraries for the GLP1-R [30,31]. As the H_4_R is predominantly expressed in the immune cells, we have used the MYTH assay to screen a Jurkat T cell Dualmembrane cDNA library for H_4_R interactors, which yielded 43 potential GIPs. Remarkably, known H_4_R interactors such as Gα_i/o_ proteins, GRKs, and β-arrestins were not retrieved in this MYTH screen. This might be related to the fact that an inactive, unliganded H_4_R conformation was used as bait in yeast cells, whereas an active (agonist-bound) H_4_R conformation is required to interact with these proteins, and additional phosphorylation of the H_4_R C-terminal tail in the case of β-arrestins1/2 [14]. Indeed, GRKs and β-arrestins have not been reported MYTH screens with ~50 GPCRs as bait, whereas only few GPCRs showed interaction with Gα_s_ (i.e., cysteinyl leukotriene receptor 2 (CYSLTR2), muscarinic acetylcholine receptor M3 (M3), Relaxin-3 receptor 1 (SALPR), and glucagon-like peptide 1 receptor (GLP1R)) and Gα_12_ (i.e., type-1 angiotensin II receptor (AGTR1)) but not the other Gα subtypes in these screens [27,29,30,31]. The unliganded H_4_R bait identified 11 putative GIPs from the Jurkat T cell DUALmembrane cDNA library that were previously observed, but not further validated, in human fetal brain DUALmembrane cDNA library for other GPCRs (Figure 1C and Supplementary Table S1) [29]. Two of these H_4_R putative hits ATP6AP2 and CD63 were also found to interact with GLP1R in human pancreatic and liver islets cDNA libraries MYTH screens, respectively, whereas six other interactors were only shared with GLP1R but none of the other tested GPCRs (Figure 1C and Supplementary Table S1) [30,31]. The interaction of SERP1 and ATP6AP2 with GLP1R were confirmed by co-immunoprecipitation experiments, and found to be important for N-linked glycosylation in HEK293 cells and GLP1-induced Ca^2+^ influx and insulin secretion in INS-1 beta cell lines, respectively [30,31]. Interestingly, CD63 has been earlier identified in a lentiviral cDNA library screen that made T cells resistant to human immunodeficiency virus (HIV)-induced cell death [47]. To this end, CD63 prevented viral entry by targeting the HIV co-receptor CXCR4 from the Golgi apparatus to late endosomes instead of cell surface [48]. In addition, CD63-mediated downregulation of CXCR4 in activated B cells was found to be essential for their migration from the dark into the light zone of germinal centers in secondary lymphoid organs formation [49]. Indeed, interaction of CD63 and CXCR4 was confirmed by co-immunoprecipitation and BiFC in transfected cells [48,50]. CD63 belongs to the structurally conserved tetraspanin family of 33 membrane-associated proteins in humans consisting of four transmembrane helices with an intracellular N- and C-termini, one intracellular loop, and two extracellular loops. In this study, H_4_R was also found to interact with TSPAN4, another tetraspanin family member, belonging to a different subfamily (i.e., CD versus CD63 subfamilies, respectively) based on sequence conservation [43]. TSPAN4 was also found to interact with the leukotriene B4 receptor 2 (LTB4R2) in a MYTH screen of human fetal brain DUALmembrane cDNA library, but was not further validated [29]. In the same study, five other tetraspanin family members (i.e., TSPAN3, TSPAN27, TSPAN33, and in particular TSPAN7 and TSPAN28) were also found to interact with a number of GPCRs (i.e., TSPAN3 with three GPCRs, TSPAN7 with 12 GPCRs, TSPAN27 with two GPCRs, TSPAN28 with 12 GPCRs, and TSPAN33 with one GPCR), but again no validation in mammalian cell lines was provided [29]. Considering that tetraspanins have been recognized as regulators of cellular signaling with a potential for therapeutical targeting [42,43], we decided to focus our validation on TSPAN4. Interestingly, the interaction between H_4_R and TSPAN4 was slightly reduced (<5–10%) in both MYTH yeast growth and BRET assays in HEK293T cells by histamine stimulation. In contrast, no effect was observed of TSPAN4 overexpression on histamine binding or H_4_R-mediated G_i_ protein activation in response to histamine. Although we cannot exclude that a fraction of H_4_R might not be in complex with TSPAN4 in these cells, the ratios between H_4_R- and TSPAN4-expressing plasmids that were used in the binding and G protein activation corresponds to approximately 80–90% and 60–70% of the saturation BRET maximum plateaus (Figure 3C,D), suggesting that the majority of H_4_R was indeed in complex with TSPAN4. Instead of overexpression studies, future research might use CRISPR/Cas9 genomic editing to inhibit TSPAN4 (and/or CD63) expression in immune cell lines to shed more light on the potential role of tetraspanins in the regulation of H_4_R functioning, or vice versa. Interestingly, TSPAN4 was recently reported to play a role in the formation of migrasomes on retracting fibers at the rear end of migrating cells [51,52]. Migrasomes are extracellular vesicles that contain signaling molecules and can be taken up by other cells to mediate cell-to-cell communication. Importantly, migrasome formation requires cell migration and the H_4_R has been reported to mediate chemotaxis of various immune cells towards histamine [9,11,53,54,55,56,57,58,59]. Hence, it is tempting to speculate whether histamine might stimulate migrasome generation by reducing the interaction between H_4_R and TSPAN4, so that the latter can be enriched in the migrasomes.

The H_4_R is also expressed in the brain and a bioinformatics approach was previously used to predict brain-specific H_4_R interacting proteins [60]. The integration of Knowledgegram and Predictogram computational analyses revealed 15 potential brain-specific GIPs for the H_4_R, which did not overlap with the potential GIPs in the here reported MYTH screen on Jurkat T cell cDNA. Experimental confirmation of these predicted brain-specific H_4_R GIPs by performing for example MYTH on brain cDNA might reveal cell type-specific regulation of H_4_R functioning.

In conclusion, our MYTH screen using H_4_R as bait on Jurkat T cell cDNA library identified 43 novel interactors, of which 17 have been previously reported for other GPCRs in MYTH screens on different cDNA libraries. These MYTH datasets might collectively provide valuable clues in the role of GIPs in regulating GPCR activities, but do require experimental validation for example by analyzing the consequences of GIP knockdown using CRISPR/Cas9 or using this genomic editing approach in combination with sensitive biosensors (e.g., NanoBiT) to measure the dynamics of these interactions at physiologically relevant expression levels [61].

## Figures and Tables

**Table 1:** Binding affinity of [^3^H]histamine for H_4_R in cell homogenates in the absence or presence of TSPAN4. Data are displayed as mean ± SD from 5 independent experiments performed in duplicate.

(co-)Expressed Constructs	pK_d_	B_max_ (fmol/mg)
H_4_R	7.9 ± 0.1	430 ± 74
H_4_R + mVenus-TSPAN4	8.0 ± 0.3	86 ± 40
H_4_R + TSPAN4-mVenus	8.0 ± 0.4	75 ± 43

## Data Availability

Not applicable.

## Supplementary Materials

The following are available online at https://www.mdpi.com/article/10.3390/biom11081127/s1, Table S1: Proteins identified by MYTH screen on Jurkat T cell DUALmembrane cDNA library using unliganded hH_4_R as bait. Figure S1: Validation of MYTH constructs. Figure S2: Total (black dot) and nonspecific (gray dot) binding of [^3^H]histamine to HEK293T cell homogenates transiently expressing H_4_R-Nluc (A), H_4_R-Nluc with mVenus-TSPAN4 (B), or H_4_R-Nluc with TSPAN4-mVenus (C).
