# Supplementary material for: Identification of TSPAN4 as Novel Histamine H4 Receptor Interactor
Source: Biomolecules. 2021 Jul 30;11(8):1127. doi: 10.3390/biom11081127 (PMC8394291; doi:10.3390/biom11081127)
Supplement: Supplementary file 1 [file biomolecules-11-01127-s001.zip › biomolecules-1319297-supplementary.pdf]

Identification of TSPAN4 as novel histamine H_4 receptor interactor

Xiaoyuan Ma[1], Eléonore W. E. Verweij [1], Marco Siderius[1], Rob Leurs[1], Henry F. Vischer[1]

Division of Medicinal Chemistry, Faculty of Science, Amsterdam Institute of Molecular and Life Sciences,

Vrije Universiteit Amsterdam, 1081 HZ Amsterdam, The Netherlands.

SUPPLEMENTARY MATERIALS

Supplementary Table S1. Proteins identified by MYTH screen on Jurkat T cell DUALmembrane cDNA library using unliganded hH_4R as bait.

Gene	Uniprot ID[1]	Protein	Localization[1]	Function[1]	Interaction with other GPCRs[2]
ALOX5AP	P20292	Arachidonate 5-lipoxygenase-activating protein	endoplasmic reticulum membrane; nuclear envelope	Required for leukotriene biosynthesis by ALOX5 (5-lipoxygenase). Anchors ALOX5 to the membrane. Binds arachidonic acid, and could play an essential role in the transfer of arachidonic acid to ALOX5. Binds to MK-886, a compound that blocks the biosynthesis of leukotrienes.	
ARID5B	Q14865	AT-rich interactive domain-containing protein 5B	nucleus	Transcription coactivator that binds to the 5'-AATA[CT]-3' core sequence and plays a key role in adipogenesis and liver development. Acts by forming a complex with phosphorylated PHF2, which mediates demethylation at Lys-336, leading to target the PHF2-ARID5B complex to target promoters, where PHF2 mediates demethylation of dimethylated 'Lys-9' of histone H3 (H3K9me2), followed by transcription activation of target genes. The PHF2-ARID5B complex acts as a coactivator of HNF4A in liver. Required for adipogenesis: regulates triglyceride metabolism in adipocytes by regulating expression of adipogenic genes. Overexpression leads to induction of smooth muscle marker genes, suggesting that it may also act as a regulator of smooth muscle cell differentiation and proliferation. Represses the cytomegalovirus enhancer.	
ARL6IP6	Q8N6S5	ADP-ribosylation factor-like protein 6-interacting protein 6	nucleus inner membrane	_	D2L [1]
ATP6AP2	O75787	Renin receptor	lysosomal membrane; endosome membrane; dendritic spine membrane; endoplasmic reticulum membrane; autophagosome membrane; axon	Multifunctional protein which functions as a renin, prorenin cellular receptor and is involved in the assembly of the lysosomal proton-transporting V-type ATPase (v-ATPase) and the acidification of the endo-lysosomal system; May mediate renin-dependent cellular responses by activating ERK1 and ERK2; By increasing the catalytic efficiency of renin in AGT/angiotensinogen conversion to angiotensin I, may also play a role in the renin-angiotensin system (RAS).	ADRA1A [1]; 5HT2C [1]; GLP1R [3]

BNIP3	Q12983	BCL2/adenovirus E1B 19 kDa protein-interacting protein 3	mitochondrion outer membrane; nuclear envelope; nucleus	Apoptosis-inducing protein that can overcome BCL2 suppression. May play a role in repartitioning calcium between the two major intracellular calcium stores in association with BCL2. Involved in mitochondrial quality control via its interaction with SPATA18/MIEAP: in response to mitochondrial damage, participates in mitochondrial protein catabolic process (also named MALM) leading to the degradation of damaged proteins inside mitochondria. The physical interaction of SPATA18/MIEAP, BNIP3 and BNIP3L/NIX at the mitochondrial outer membrane regulates the opening of a pore in the mitochondrial double membrane in order to mediate the translocation of lysosomal proteins from the cytoplasm to the mitochondrial matrix. Plays an important role in the calprotectin (S100A8/A9)-induced cell death pathway.	GPR37 [1]; HTR2B [1]; AGTR1 [1]
CCDC167	Q9P0B6	Coiled-coil domain-containing protein 167	membrane	_	GLP1R [3]
CD63	P08962	CD63 antigen	endosome membrane, plasma membrane, extracellular exosome, lysosome membrane, melanosome, nucleus	Functions as cell surface receptor for TIMP1 and plays a role in the activation of cellular signaling cascades. Plays a role in the activation of ITGB1 and integrin signaling, leading to the activation of AKT, FAK/PTK2 and MAP kinases. Promotes cell survival, reorganization of the actin cytoskeleton, cell adhesion, spreading and migration, via its role in the activation of AKT and FAK/PTK2. Plays a role in VEGFA signaling via its role in regulating the internalization of KDR/VEGFR2. Plays a role in intracellular vesicular transport processes, and is required for normal trafficking of the PMEL luminal domain that is essential for the development and maturation of melanocytes. Plays a role in the adhesion of leukocytes onto endothelial cells via its role in the regulation of SELP trafficking. May play a role in mast cell degranulation in response to Ms4a2/FceRI stimulation, but not in mast cell degranulation in response to other stimuli.	F2RL1 [1]; GLP1R [4]
CEP20	Q96NB1	Centrosomal protein 20	centriole; cilium basal body; centrosome; centriolar satellite; cilium; cytoplasmic granule; nucleus	Involved in the biogenesis of cilia; Required for the recruitment of PLK1 to centrosomes and S phase progression.	
CERS2	Q96G23	Ceramide synthase 2	endoplasmic reticulum membrane	Ceramide synthase that catalyzes formation of ceramide from sphinganine and acyl-CoA substrates, with high selectivity toward very-long (C22:0-C24:0) chain as acyl donor; May regulate lipid metabolism in hepatocytes.	D2L [1]
CHIC2	Q9UKJ5	Cysteine-rich hydrophobic domain-containing protein 2	plasma membrane; cytoplasmic vesicle	_	
CNIH1	O95406	Protein cornichon homolog 1	Golgi apparatus membrane; endoplasmic reticulum membrane	Involved in the selective transport and maturation of TGF-alpha family proteins.	
COX8A	P10176	Cytochrome c oxidase subunit 8A, mitochondrial	inner mitochondrial membrane	Component of the cytochrome c oxidase, the last enzyme in the mitochondrial electron transport chain which drives oxidative phosphorylation. The respiratory chain contains 3 multisubunit complexes succinate dehydrogenase (complex II, CII), ubiquinol-cytochrome c oxidoreductase (cytochrome b-c1 complex, complex III, CIII) and cytochrome c oxidase (complex IV, CIV), that cooperate to transfer electrons derived from NADH and succinate to molecular oxygen, creating an electrochemical gradient over the inner membrane that drives transmembrane transport and the ATP synthase. Cytochrome c oxidase is the component of the respiratory chain that catalyzes the reduction of oxygen to water. Electrons originating from reduced cytochrome c in the intermembrane space (IMS) are transferred via the dinuclear copper A center (CU(A)) of subunit 2 and heme A of subunit 1 to the active site in subunit 1, a binuclear center (BNC) formed by heme A3 and copper B (CU(B)). The BNC reduces molecular oxygen to 2 water molecules using 4 electrons from cytochrome c in the IMS and 4 protons from the mitochondrial matrix.	

EBP	Q15125	3-beta-hydroxysteroid-Delta(8),Delta(7)-isomerase	endoplasmic reticulum membrane; nuclear envelope; cytoplasmic vesicle	Catalyzes the conversion of Delta8-sterols to their corresponding Delta7-isomers.	GLP1R [4]
ECH1	Q13011	Delta(3,5)-Delta(2,4)-dienoyl-CoA isomerase, mitochondrial	peroxisome; mitochondrion	Isomerization of 3-trans,5-cis-dienoyl-CoA to 2-trans,4-trans-dienoyl-CoA.	
EMC7	Q9NPA0	ER membrane protein complex subunit 7	endoplasmic reticulum membrane	Part of the endoplasmic reticulum membrane protein complex (EMC) that enables the energy-independent insertion into endoplasmic reticulum membranes of newly synthesized membrane proteins; Preferentially accommodates proteins with transmembrane domains that are weakly hydrophobic or contain destabilizing features such as charged and aromatic residues; Involved in the cotranslational insertion of multi-pass membrane proteins in which stop-transfer membrane-anchor sequences become ER membrane spanning helices; It is also required for the post-translational insertion of tail-anchored/TA proteins in endoplasmic reticulum membranes.	GLP1R [4]
FAM168A	Q92567	Protein FAM168A	_	In cancer context, protects cells from induced-DNA damage and apoptosis; Acts, at least in part, through PI3K/AKT/NFKB signaling pathway and by preventing POLB degradation; Decreases POLB ubiquitation and stabilizes its protein levels.	
FXYD5	Q96DB9	FXYD domain-containing ion transport regulator 5	membrane	Involved in down-regulation of E-cadherin which results in reduced cell adhesion; Promotes metastasis.	
IER3IP1	Q9Y5U9	Immediate early response 3-interacting protein 1	endoplasmic reticulum membrane	Regulator of endoplasmic reticulum secretion that acts as a key determinant of brain size; Required for secretion of extracellular matrix proteins; Required for correct brain development by depositing sufficient extracellular matrix proteins for tissue integrity and the proliferation of neural progenitors.	
ITGB1	P05556	Integrin beta-1	recycling endosome; plasma membrane; invadopodium membrane; ruffle membrane; melanosome; Cleavage furrow; lamellipodium; focal adhesion	ITGB1 acts as a receptor for fibrillin-1 (FBN1) and mediates R-G-D-dependent cell adhesion to FBN1; ITGB1 is a receptor for IL1B and binding is essential for IL1B signaling.	
KDELR1	P24390	ER lumen protein-retaining receptor 1	endoplasmic reticulum membrane; Golgi apparatus membrane; COPI-coated vesicle membrane; endoplasmic reticulum-Golgi intermediate compartment membrane	Receptor for the C-terminal sequence motif K-D-E-L that is present on endoplasmic reticulum resident proteins and that mediates their recycling from the Golgi back to the endoplasmic reticulum.	CCR9 [1]; F2RL1 [1]; GPR35 [1]
LAPTM4B	Q86VI4	Lysosomal-associated transmembrane protein 4B	plasma membrane; endosome membrane; multivesicular body membrane; multivesicular	Blocks EGF-stimulated EGFR intraluminal sorting and degradation. Conversely by binding with the phosphatidylinositol 4,5-bisphosphate, regulates its PIP5K1C interaction, inhibits HGS ubiquitination and relieves LAPTM4B inhibition of EGFR degradation; Recruits SLC3A2 and SLC7A5 (the Leu transporter) to the lysosome, promoting entry of leucine and other essential amino acid (EAA) into the lysosome, stimulating	BDKRB1 [1]

			body lumen; lysosomal membrane.	activation of proton-transporting vacuolar (V)-ATPase protein pump (V-ATPase) and hence mTORC1 activation; Plays a role as negative regulator of TGFB1 production in regulatory T cells.	
MSMO1	Q15800	Methylsterol monooxygenase 1	endoplasmic reticulum membrane	Catalyzes the three-step monooxygenation required for the demethylation of 4,4-dimethyl and 4alpha-methylsterols, which can be subsequently metabolized to cholesterol; Also involved in drug metabolism, as it can metabolize eldecalcitol (ED-71 or 1alpha,25-dihydroxy-2beta-(3-hydroxypropoxy)-cholecalciferol), a second-generation vitamin D analog, into 1alpha,2beta,25-trihydroxy vitamin D3; this reaction occurs via enzymatic hydroxylation and spontaneous O-dehydroxypropylation.	GPR35 [1]; M5 [1]
MT-ATP6	P00846	ATP synthase subunit a	inner mitochondrial membrane	Mitochondrial membrane ATP synthase (F1F0 ATP synthase or Complex V) produces ATP from ADP in the presence of a proton gradient across the membrane which is generated by electron transport complexes of the respiratory chain. F-type ATPases consist of two structural domains, F1 - containing the extramembraneous catalytic core and F0 - containing the membrane proton channel, linked together by a central stalk and a peripheral stalk. During catalysis, ATP synthesis in the catalytic domain of F1 is coupled via a rotary mechanism of the central stalk subunits to proton translocation. Key component of the proton channel; it may play a direct role in the translocation of protons across the membrane.	GLP1R [4]
MT-CO2	P00403	Cytochrome c oxidase subunit 2	inner mitochondrial membrane	Component of the cytochrome c oxidase, the last enzyme in the mitochondrial electron transport chain which drives oxidative phosphorylation. The respiratory chain contains 3 multisubunit complexes succinate dehydrogenase (complex II, CII), ubiquinol-cytochrome c oxidoreductase (cytochrome b-c1 complex, complex III, CIII) and cytochrome c oxidase (complex IV, CIV), that cooperate to transfer electrons derived from NADH and succinate to molecular oxygen, creating an electrochemical gradient over the inner membrane that drives transmembrane transport and the ATP synthase. Cytochrome c oxidase is the component of the respiratory chain that catalyzes the reduction of oxygen to water. Electrons originating from reduced cytochrome c in the intermembrane space (IMS) are transferred via the dinuclear copper A center (CU(A)) of subunit 2 and heme A of subunit 1 to the active site in subunit 1, a binuclear center (BNC) formed by heme A3 and copper B (CU(B)). The BNC reduces molecular oxygen to 2 water molecules using 4 electrons from cytochrome c in the IMS and 4 protons from the mitochondrial matrix.	
MT-CO3	P00414	Cytochrome c oxidase subunit 3	inner mitochondrial membrane	Component of the cytochrome c oxidase, the last enzyme in the mitochondrial electron transport chain which drives oxidative phosphorylation. The respiratory chain contains 3 multisubunit complexes succinate dehydrogenase (complex II, CII), ubiquinol-cytochrome c oxidoreductase (cytochrome b-c1 complex, complex III, CIII) and cytochrome c oxidase (complex IV, CIV), that cooperate to transfer electrons derived from NADH and succinate to molecular oxygen, creating an electrochemical gradient over the inner membrane that drives transmembrane transport and the ATP synthase. Cytochrome c oxidase is the component of the respiratory chain that catalyzes the reduction of oxygen to water. Electrons originating from reduced cytochrome c in the intermembrane space (IMS) are transferred via the dinuclear copper A center (CU(A)) of subunit 2 and heme A of subunit 1 to the active site in subunit 1, a binuclear center (BNC) formed by heme A3 and copper B (CU(B)). The BNC reduces molecular oxygen to 2 water molecules using 4 electrons from cytochrome c in the IMS and 4 protons from the mitochondrial matrix.	
NAPG	Q99747	Gamma-soluble NSF attachment protein	lysosomal membrane; mitochondrion	Required for vesicular transport between the endoplasmic reticulum and the Golgi apparatus.	
NRM	Q8IXM6	Nurim	nucleus inner membrane.	_	
OST4	P0C6T2	Dolichyl-diphosphooligosaccharide--protein glycosyltransferase subunit 4	endoplasmic reticulum membrane	Subunit of the oligosaccharyl transferase (OST) complex that catalyzes the initial transfer of a defined glycan (Glc3Man9GlcNAc2 in eukaryotes) from the lipid carrier dolichol-pyrophosphate to an asparagine residue within an Asn-X-Ser/Thr consensus motif in nascent polypeptide chains, the first step in protein N-	GLP1R [3]

				glycosylation. N-glycosylation occurs cotranslationally and the complex associates with the Sec61 complex at the channel-forming translocon complex that mediates protein translocation across the endoplasmic reticulum (ER). All subunits are required for a maximal enzyme activity. Specifically involved in maintaining stability of STT3A-containing OST complexes.	
RNASEK	Q6P5S7	Ribonuclease kappa	membrane	Endoribonuclease which preferentially cleaves ApU and ApG phosphodiester bonds. Hydrolyzes UpU bonds at a lower rate.; Required for the initial stages of clathrin-mediated endocytic uptake of a diverse set of viruses, including dengue, West Nile, Sindbis, Rift Valley Fever, and influenza viruses.	CYSLTR2 [1]; TAAR1 [1]
SERP1	Q9Y6X1	Stress-associated endoplasmic reticulum protein 1	endoplasmic reticulum membrane	Interacts with target proteins during their translocation into the lumen of the endoplasmic reticulum. Protects unfolded target proteins against degradation during ER stress. May facilitate glycosylation of target proteins after termination of ER stress. May modulate the use of N-glycosylation sites on target proteins	GLP1R [4]
SLAIN2	Q9P270	SLAIN motif-containing protein 2	cytoskeleton.	Binds to the plus end of microtubules and regulates microtubule dynamics and microtubule organization. Promotes cytoplasmic microtubule nucleation and elongation; Required for normal structure of the microtubule cytoskeleton during interphase.	
SLC25A3	Q00325	Phosphate carrier protein, mitochondrial	inner mitochondrial membrane	Transport of phosphate groups from the cytosol to the mitochondrial matrix. Phosphate is cotransported with H+. May play a role regulation of the mitochondrial permeability transition pore (mPTP).	
SLC50A1	Q9BRV3	Sugar transporter SWEET1	plasma membrane; Golgi apparatus membrane	Mediates sugar transport across membranes; May stimulate V(D)J recombination by the activation of RAG1.	
SOX4	Q06945	Transcription factor SOX-4	nucleus	Transcriptional activator that binds with high affinity to the T-cell enhancer motif 5'-AACAAAG-3' motif.	
SSR3	Q9UNL2	Translocon-associated protein subunit gamma	endoplasmic reticulum membrane	TRAP proteins are part of a complex whose function is to bind calcium to the ER membrane and thereby regulate the retention of ER resident proteins.	
TMEM128	Q5BJH2	Transmembrane protein 128	membrane	_	
TMEM14A	Q9Y6G1	Transmembrane protein 14A	endoplasmic reticulum membrane; mitochondrial membrane	Inhibits apoptosis via negative regulation of the mitochondrial outer membrane permeabilization involved in apoptotic signaling pathway.	
TMEM230	Q96A57	Transmembrane protein 230	endosome membrane; trans-Golgi network; synaptic vesicle; autophagosome	Involved in trafficking and recycling of synaptic vesicles.	HTR4 [1]
TMEM41B	Q5BJD5	Transmembrane protein 41B	endoplasmic reticulum membrane; cytoplasm	Required for autophagosome formation; Participates in early stages of autophagosome biogenesis at the ER membrane probably via mobilization of neutral lipids from lipid droplets; Required for normal motor neuron development.	
TMEM86A	Q8N2M4	Lysoplasmalogenase-like protein TMEM86A	membrane	alkenylglycerophosphocholine hydrolase activity.	
TSPAN4	O14817	Tetraspanin-4	plasma membrane	Antigen binding; Integrin binding; Protein-containing complex assembly.	LTB4R2 [1]

ZDHHC12	Q96GR4	Palmitoyltransferase ZDHHC12	endoplasmic reticulum membrane; Golgi apparatus membrane	Palmitoyltransferase that could catalyze the addition of palmitate onto various protein substrates; Has a palmitoyltransferase activity toward gephyrin/GPHN, regulating its clustering at synapses and its function in gamma-aminobutyric acid receptor clustering. Thereby, indirectly regulates GABAergic synaptic transmission.	
ZNF714	Q96N38	Zinc finger protein 714	nucleus	May be involved in transcriptional regulation.	

[1]Protein information was retrieved from Uniprot database (https://www.uniprot.org ; accessed 24 June 2021) [2]

[2]Interactions identified with other GPCR subtypes (gene names are provided) identified in previously reported MYTH screens.

References Table S1

1. Sokolina, K.; Kittanakom, S.; Snider, J.; Kotlyar, M.; Maurice, P.; Gandía, J.; Benleulmi-Chaachoua, A.; Tadagaki, K.; Oishi, A.; Wong, V.; Malty, R. H.; Deineko, V.; Aoki, H.; Amin, S.; Yao, Z.; Morató, X.; Otasek, D.; Kobayashi, H.; Menendez, J.; Auerbach, D.; Angers, S.; Pržulj, N.; Bouvier, M.; Babu, M.; Ciruela, F.; Jockers, R.; Jurisica, I.; Stagljar, I. Systematic protein-protein interaction mapping for clinically relevant human GPCRs. *Mol. Syst. Biol.* **2017**, *13*, 918.
2. UniProt Consortium UniProt: the universal protein knowledgebase in 2021. *Nucleic Acids Res* **2021**, *49*, D480–D489.
3. Dai, F. F.; Bhattacharjee, A.; Liu, Y.; Batchuluun, B.; Zhang, M.; Wang, X. S.; Huang, X.; Luu, L.; Zhu, D.; Gaisano, H.; Wheeler, M. B. A Novel GLP1 Receptor Interacting Protein ATP6ap2 Regulates Insulin Secretion in Pancreatic Beta Cells. *Journal of Biological Chemistry* **2015**, *290*, 25045–25061.
4. Xiao, Y.; Han, J.; Wang, Q.; Mao, Y.; Wei, M.; Jia, W.; Wei, L. A Novel Interacting Protein SERP1 Regulates the N-Linked Glycosylation and Function of GLP-1 Receptor in the Liver. *J Cell Biochem* **2017**, *118*, 3616–3626.

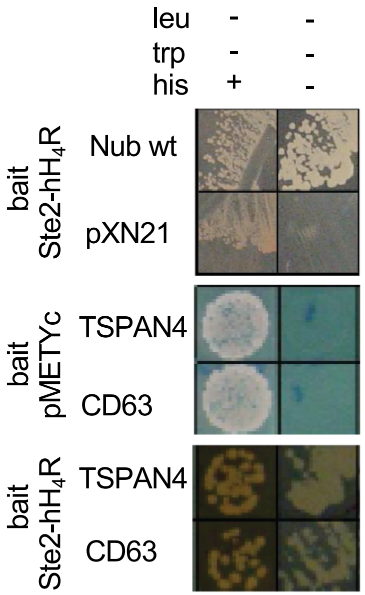

Figure S1. Validation of MYTH constructs. Diploids were generated carrying the indicated bait and prey plasmids and growth on YNB-selective media was assessed. Nub wt is the non-mutated positive control for ubiquitin reconstitution, whereas pXN21 is the negative control. NubG-hTSPAN4 and NubG-hCD63 were only capable to sustain growth when expressed together with hH_4R-Cub-LexA-VP16 (ste2-hH_4R) but not with the negative control pMETYc.

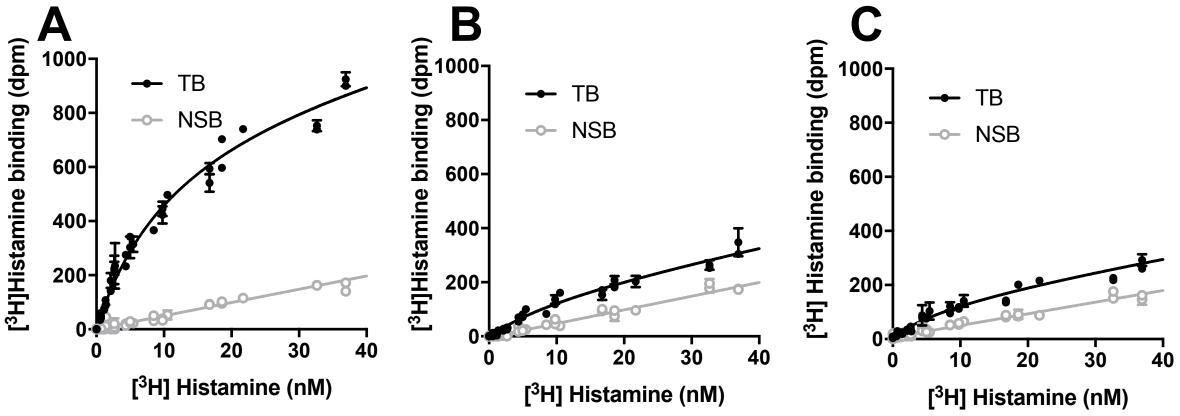

Figure S2. Total (black dot) and nonspecific (gray dot) binding of [^{3}H]histamine to HEK293T cell homogenates transiently expressing H_4R-Nluc (A), H_4R-Nluc with mVenus-TSPAN4 (B), or H_4R-Nluc with TSPAN4-mVenus (C). Data are displayed as mean ± SEM from 5 independent experiments performed in duplicate.
